# Supplementary material for: Nuclear transporter Importin-13 plays a key role in the oxidative stress transcriptional response
Source: Nat Commun. 2021 Oct 8;12:5904. doi: 10.1038/s41467-021-26125-x (PMC8501021; doi:10.1038/s41467-021-26125-x)
Supplement: Supplementary file 1 — Supplementary Information [file 41467_2021_26125_MOESM1_ESM.pdf]

Supplementary Information

Nuclear transporter Importin-13 plays a key role in the oxidative stress transcriptional response

Gajewska, K. A, Lescesen, H, Ramialison, M, Wagstaff, K. M, Jans, D. A.

Supplementary Figures

a

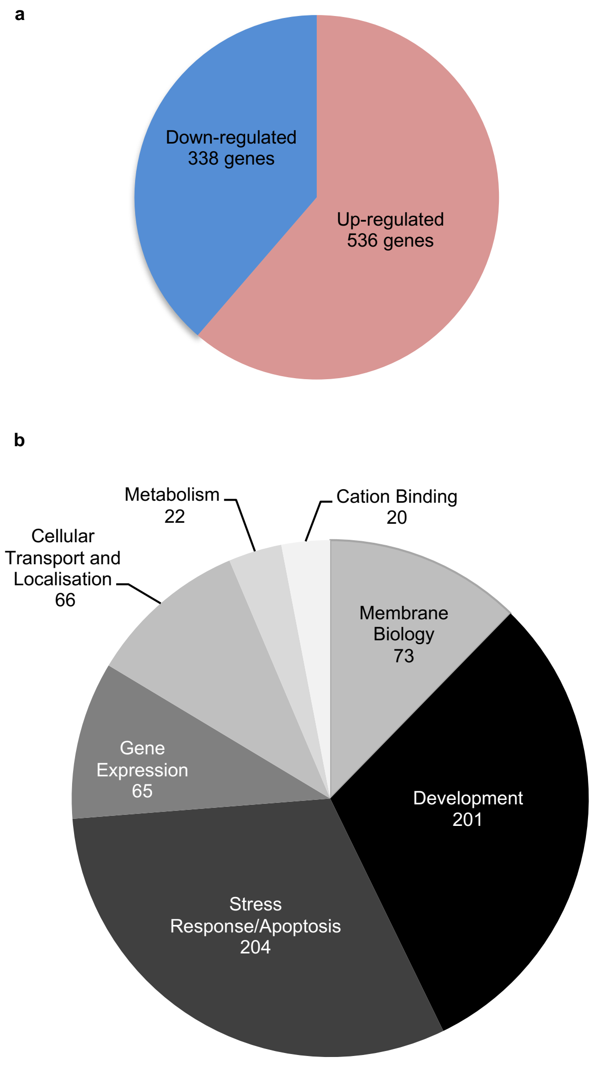

Supplementary Figure 1. The IPO13-dependent transcriptome in ESCs. Untreated $IPO13^{+/+}$ and $IPO13^{-/-}$ ESCs were subjected to NGS as described in the Results section. **a.** Log2-fold change in expression (FDR cut-off = 0.05) of genes in $IPO13^{-/-}$ compared to $IPO13^{+/+}$ ESCs are shown, where red and blue denote up- and down-regulated genes respectively. **b.** Gene Ontology analysis of differentially expressed genes in **a** using the Functional Annotation Tool of DAVID Bioinformatics Resources 6.8 for 651 annotated genes.

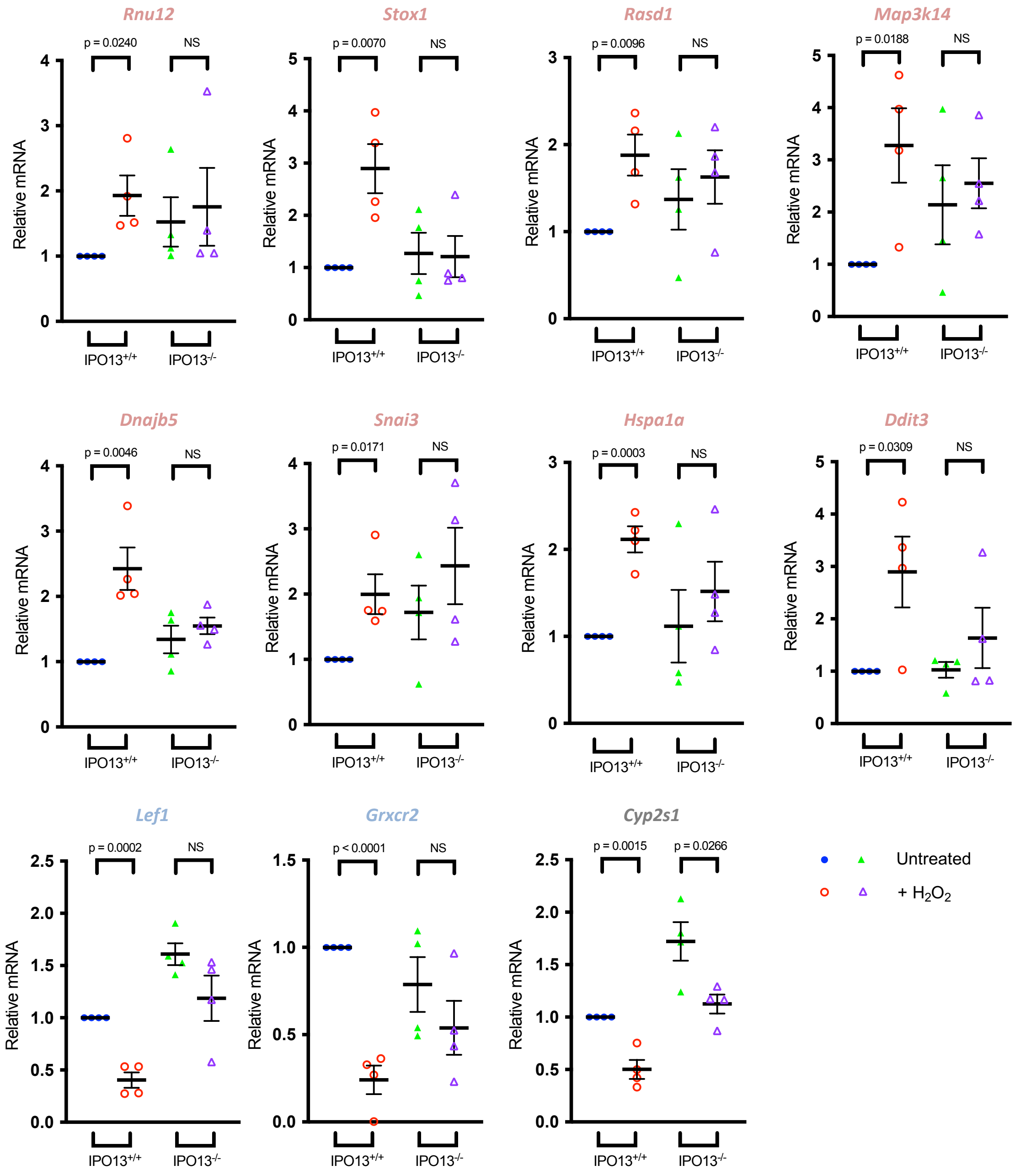

Supplementary Figure 2. RT-qPCR validation of selected genes showing IPO13-dependent stress responses. $IPO13^{+/+}$ and $IPO13^{-/-}$ ESCs were treated without and with 125 μM H_2O_2 for 1 h followed by 2 h recovery prior to RNA extraction. Transcript levels were assessed using SensiMix SYBR Green (Bioline Reagents, London, UK) on a C1000 Touch Thermal Cycler (Biorad). After normalization of gene expression relative to the expression of housekeeping genes *Sdha* and *Tbp*, data were relativized to untreated $IPO13^{+/+}$. Gene names in red and blue denote those up- or down-regulated respectively in response to stress in $IPO13^{+/+}$ ESCs, with the gene name in grey denoting a gene down-regulated in response to stress in both $IPO13^{+/+}$ and $IPO13^{-/-}$ ESCs. Results represent the mean ± SEM (n = 4, where 2 cDNA samples were measured in duplicate). p values (two tailed student's t test) left to right top to bottom: p = 0.0240, p = 0.7544, p = 0.0070, p = 0.9166, p = 0.0096, p = 0.5994, p = 0.0188, p = 0.6615, p = 0.0046, p = 0.4313, p = 0.0171, p = 0.3593, p = 0.0003, p= 0.4878, p = 0.0309, p = 0.3467, p = 0.0002, p = 0.1306, p < 0.0001, p = 0.3048, p = 0.0015 and p = 0.0266. Source data are provided as a Source Data file.

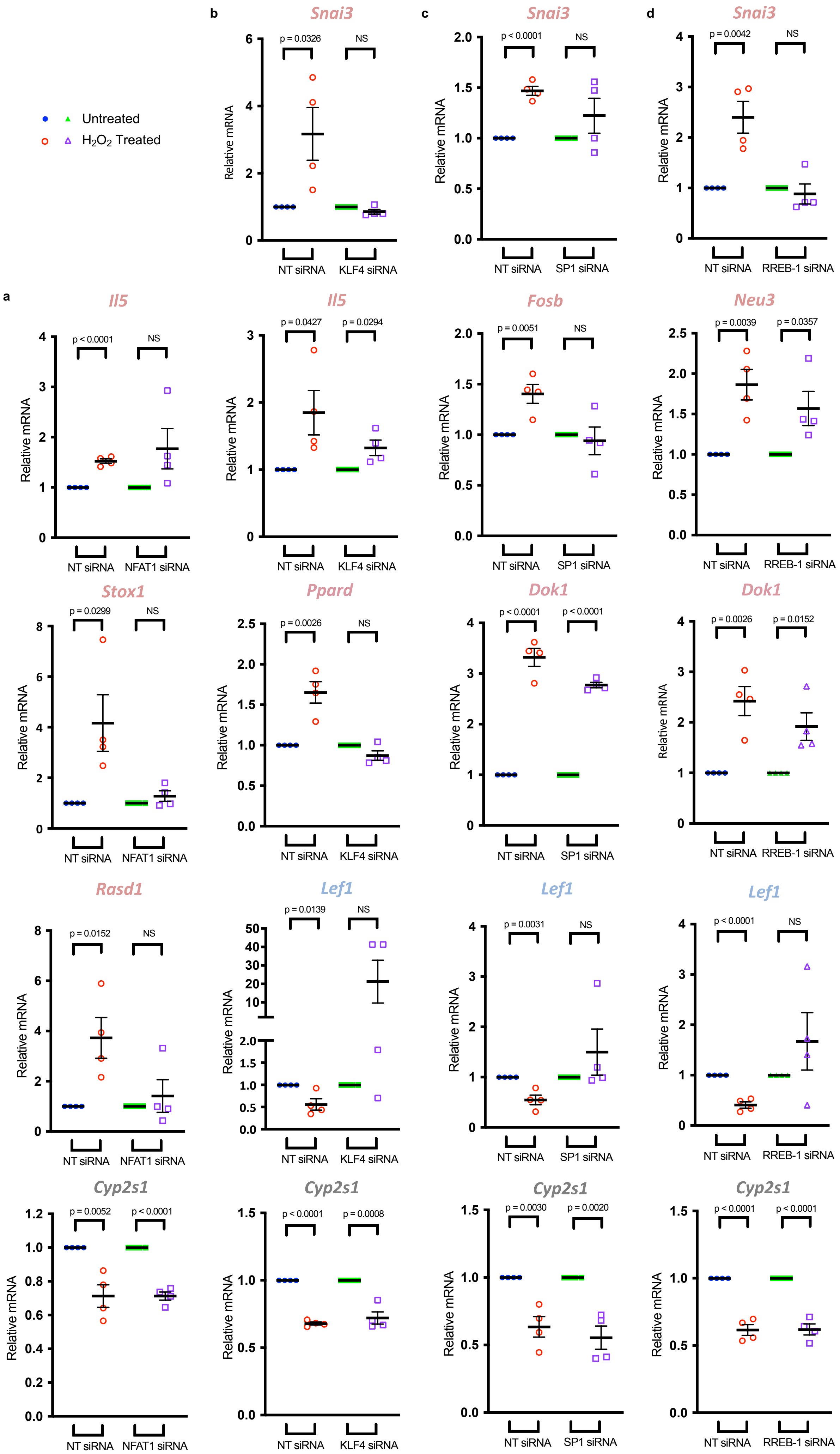

a
b
c
d
Untreated
H2O2 Treated
Snai3
Il5
Fosb
Neu3
Stox1
Ppard
Dok1
Rasd1
Lef1
Cyp2s1
Relative mRNA
NT siRNA
NFAT1 siRNA
KLF4 siRNA
SP1 siRNA
RREB-1 siRNA
p = 0.0326
p < 0.0001
p = 0.0042
p = 0.0427
p = 0.0294
p = 0.0051
p = 0.0039
p = 0.0357
p = 0.0299
p = 0.0026
p = 0.0152
p = 0.0139
p = 0.0031
p = 0.0052
p = 0.0008
p = 0.0030
p = 0.0020
NS

e

f

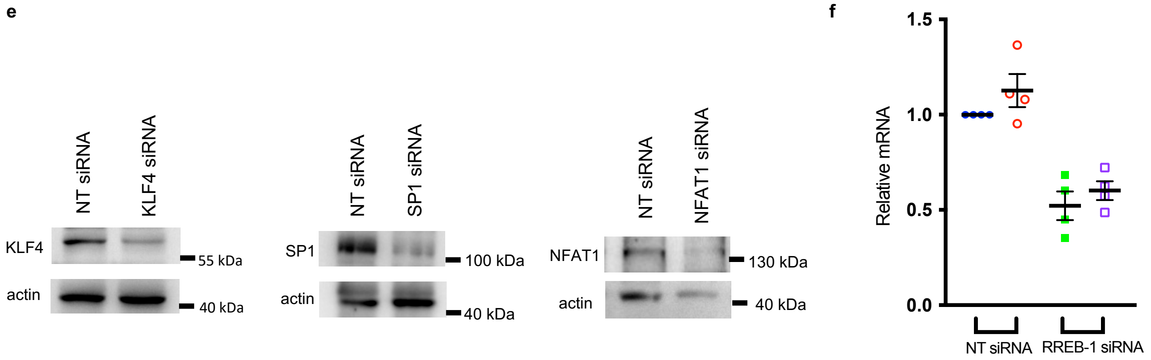

Supplementary Figure 3. RT-qPCR analysis of expression changes regulated in response to oxidative stress in genes containing TF consensus motifs. RT-qPCR analysis of genes treated to oxidative stress. IPO13$^{+/+}$ ESCs were treated with siRNA targeting **a.** NFAT1, **b.** KLF4, **c.** SP1, **d.** RREB-1 or non-targeting (NT) siRNA**.** 3 d post transfection, cells were treated with 125 μM H_2O_2 for 1 h followed by a 2 h recovery prior to RNA extraction. Transcript levels were assessed as per Sup. Fig. 2 legend. Gene names in red and blue denote those up- or down-regulated respectively in response to stress in IPO13$^{+/+}$ ESCs, with gene name in grey denoting a gene down-regulated in response to stress in both IPO13$^{+/+}$ and IPO13$^{-/-}$ ESCs as per Supplementary Figure 2. After normalization of gene expression relative to the expression of housekeeping genes *Sdha* and *Tbp*, data were relativized to the respective basal gene expression values for specific/NT siRNA respectively. qPCR data represents the mean ± SEM (n = 4, where 2 cDNA samples were measured in duplicate). p values (two tailed student's t test) top to bottom left to right **a**: $p < 0.0001$, $p = 0.1034$, $p = 0.0299$, $p = 0.2266$, $p = 0.0152$, $p = 0.7788$, $p= 0.0052$ and $p < 0.0001$, **b**: $p = 0.0326$, $p = 0.0863$, $p = 0.0427$, $p = 0.0294$, $p = 0.0026$, $p = 0.0709$, $p = 0.0139$, $p = 0.1302$, $p < 0.0001$ and $p = 0.0008$, **c**: $p < 0.0001$, $p = 0.2446$, $p = 0.0051$, $p = 0.6788$, $p < 0.0001$, $p < 0.0001$, $p = 0.0031$, $p = 0.3188$, $p = 0.0030$ and $p = 0.0020$, **d**: $p = 0.0042$, $p = 0.5770$, $p = 0.0039$, $p = 0.0357$, $p = 0.0026$, $p = 0.0152$, $p < 0.0001$, $p = 0.2816$, $p < 0.0001$ and $p < 0.0001$. Total cell extracts were probed for knockdown by Western Blotting **(e)** using rabbit-anti-KLF4 (Abcam), rabbit-anti-SP1 (Milipore) or mouse-anti-NFAT1 (Abcam), or mouse-anti-actin (Abcam) for loading control, followed by imaging using the ChemiDoc Gel Imaging System (Biorad). **f**. RT-qPCR repeated as in **d**, measuring RREB-1 transcript levels. Data represents the mean ± SEM (n = 4, where 2 cDNA samples were measured in duplicate). Source data are provided as a Source Data file.

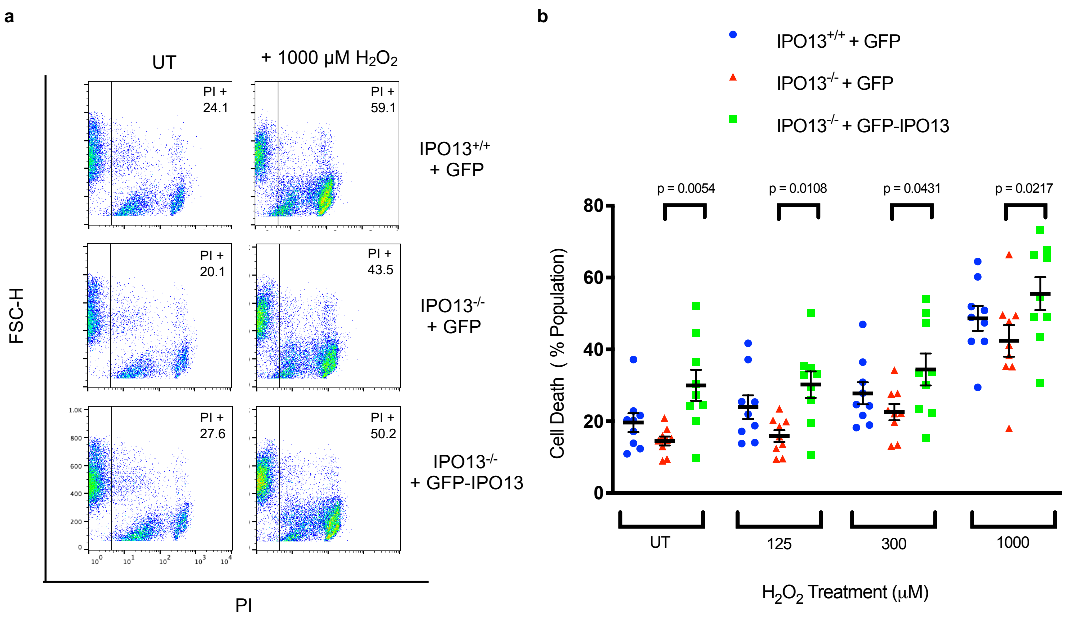

Supplementary Figure 4. Ectopic expression of IPO13 rescues IPO13$^{-/-}$ ESC susceptibility to H_2O_2-induced cell death. IPO13$^{+/+}$ ESCs were transfected with GFP and IPO13$^{-/-}$ ESCs were transfected with either GFP or GFP-tagged IPO13. ESCs were then treated with H_2O_2 as indicated for 1 h and stained with PI for flow cytometric analysis. **a.** Representative dot plots of the indicated samples with the percentage of ESC PI positive. **b.** Pooled data (n = 9 independent experiments) for % PI positive cells from the analysis such as that in **a** are shown (mean $\pm$ SEM), with p values for comparisons indicated, two-way ANOVA and Tukey's multiple comparisons test (left to right: p = 0.0054, p = 0.0108, p = 0.0431 and p = 0.0217). Source data are provided as a Source Data file.

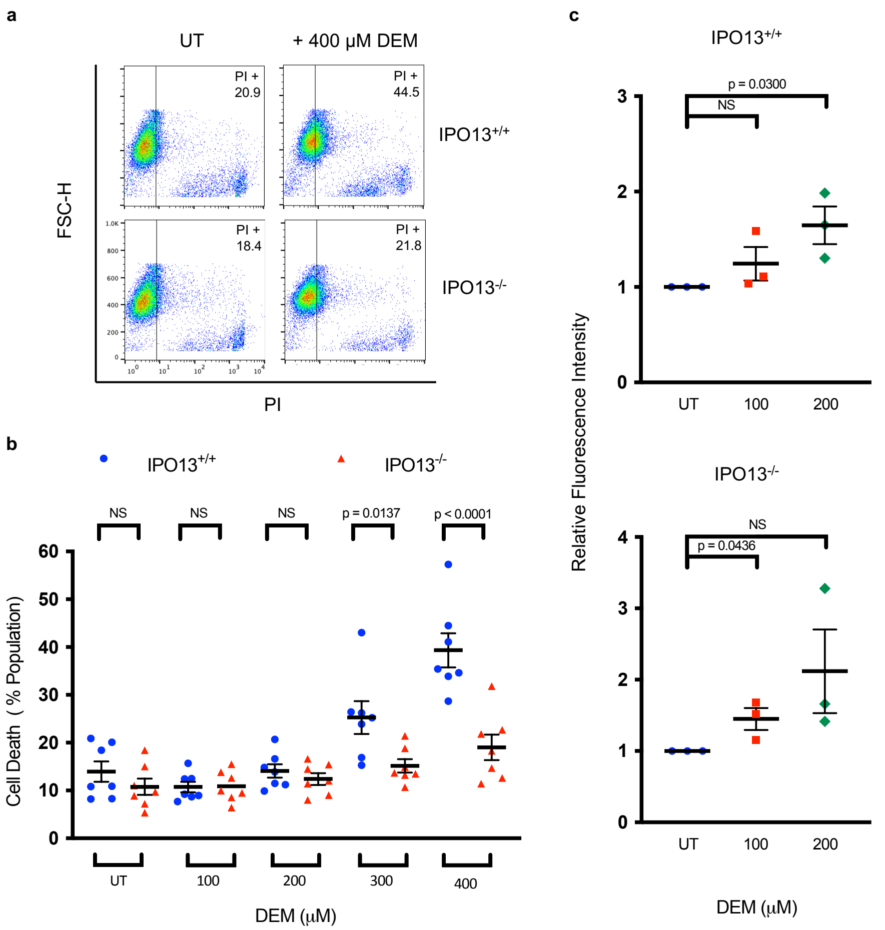

Supplementary Figure 5. IPO13 contributes to DEM-induced oxidative stress-induced cell death. IPO13$^{+/+}$ and IPO13$^{-/-}$ ESCs were treated with DEM as indicated for 24h, and then stained with PI and analysed by flow cytometry. **a.** Representative dot plots of the indicated samples with the percentage of ESC PI positive. **b.** Pooled data (n = 7 independent experiments) for % PI positive cells from the analysis such as that in **a** are shown (mean $\pm$ SEM), two-way ANOVA and Tukey's multiple comparisons test. P values from left to right: $p = 0.9730$, $p > 0.9999$, $p = 0.9998$, $p = 0.0137$ and $p < 0.0001$. **c.** IPO13$^{+/+}$ and IPO13$^{-/-}$ ESCs were treated with DEM as indicated for 24 h and then stained immediately with 5 μM CellROX Green for 30 min and analysed by flow cytometry. Quantification of the fluorescence intensity relative to the untreated (UT) sample are shown representing the mean $\pm$ SEM (n=3 independent experiments), p values (two tailed student's t test) left to right **b**: $p = 0.9730$, $p > 0.9990$, $p = 0.9998$, $p = 0.0137$, $p < 0.0001$ and top to bottom **c**: $p = 0.0300$, $p = 0.2339$, $p = 0.1287$, $p = 0.0436$. Source data are provided as a Source Data file.

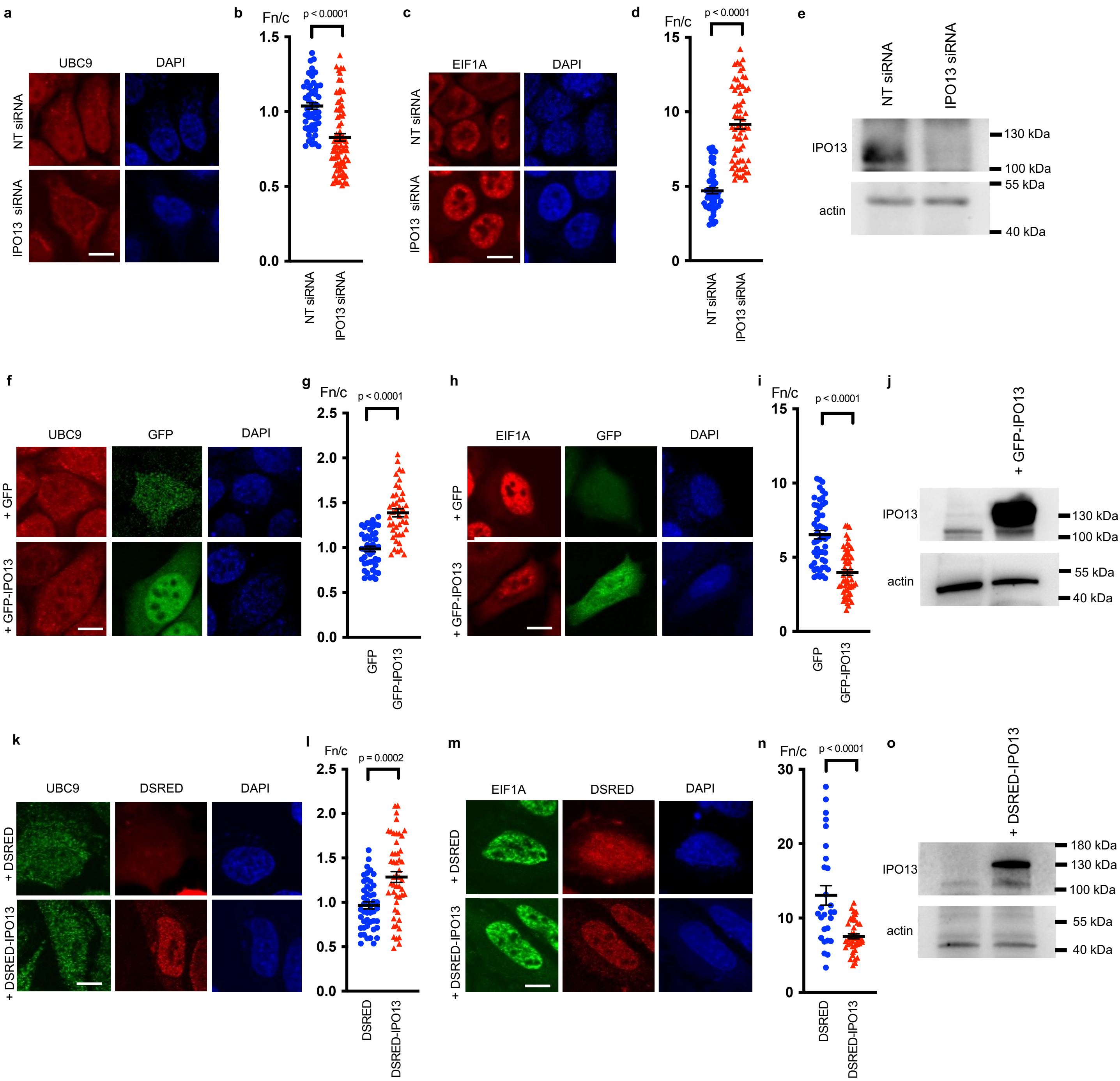

Supplementary Figure 6. Ectopic expression or knock down of IPO13 affects the localisation of IPO13 import and export cargoes. a-e. HeLa cells were transfected with non-targeting (NT) or IPO13 targeting siRNA 72 h prior to processing for CLSM imaging. All cells were counterstained with DAPI to highlight nuclei. **a**. Representative images of cells immunostained with anti-UBC9. Scale bar = 10 µm **b**. Quantitative analysis of endogenous UBC9 protein localisation was carried out using the ImageJ software on images such as those in **a**, to determine the nuclear to cytoplasmic ratio (Fn/c). Data represent the mean + SEM of the nuclear (Fn) and cytoplasmic fluorescence (Fc) above background fluorescence for 61 cells (NT) and 81 cells (IPO13 siRNA). p value (two tailed student's t test) : $p < 0.0001$. **c**. Representative images of cells immunostained with anti-EIF1A. Scale bar = 10 µm **d**. Quantitative analysis of endogenous EIF1A localisation carried out as per **b** for 50 cells (NT) and 66 cells (IPO13 siRNA). p value (two tailed student's t test): $p < 0.0001$. **e**. Total cell extracts were probed by Western blotting using rabbit-anti-IPO13 (Protein Tech), with mouse-anti-actin (Abcam) as a control, and imaged using the ChemiDoc Gel Imaging System (Biorad). **f-j.** HeLa cells were transfected to express either GFP or GFP-tagged IPO13 immediately processed for CLSM imaging and/or Western analysis. **f.** Representative images of cells immunostained with anti-UBC9. Scale bar = 10 µm **g**. Quantitative analysis of endogenous UBC9 protein localisation performed as per **b**, representative of the mean + SEM for 45 cells (GFP) and 43 cells (GFP-IPO13). p value (two tailed student's t test): $p < 0.0001$. **h**. Representative images of cells immunostained with anti-EIF1A. Scale bar = 10 µm **i**. Quantitative analysis of

endogenous EIF1A localisation carried out as per **b** for 52 cells (GFP) and 57 cells (GFP-IPO13). p value (two tailed student's t test): $p < 0.0001$. **j**. Total cell extracts were probed as in **e**. **k-o**. HeLa cells were transfected to express either DSRED or DSRED-tagged IPO13 immediately processed for CLSM imaging and/or Western analysis. **k**. Representative images of cells immunostained with anti-UBC9. Scale bar = 10 µm **l**. Quantitative analysis of endogenous UBC9 protein localisation performed as per **b**, representative of the mean ± SEM for 47 cells (DSRED) and 50 cells (DSRED-IPO13). p value (two tailed student's t test): $p = 0.0002$. **m**. Representative images of cells immunostained with anti-EIF1A. Scale bar = 10 µm **n**. Quantitative analysis of endogenous EIF1A localisation carried out as per **b**, representative of the mean ± SEM for 27 cells (DSRED) and 40 cells (DSRED-IPO13). p value (two tailed student's t test): $p < 0.0001$. **o.** Total cell extracts were probed as in **e**. Source data are provided as a Source Data file.

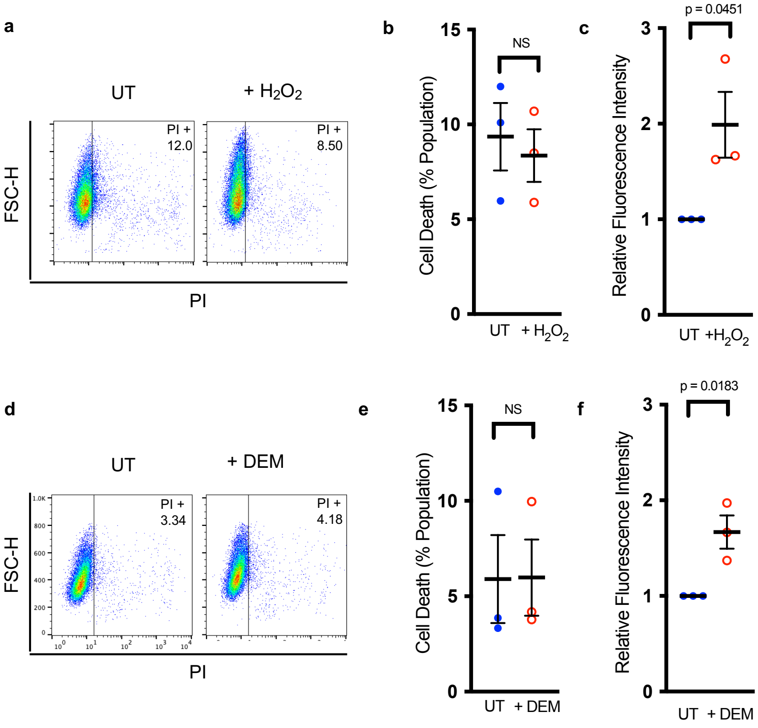

Supplementary Figure 7. Sublethal concentration of hydrogen peroxide and DEM utilised in localisation and interaction studies do not induce cell death in HeLa cells. a-b. HeLa cells were treated with 125 µM H_2O_2 for 1 h and then stained immediately with propidium iodide for flow cytometric analysis of cell death. **a.** Representative dot plots of the indicated samples with the percentage of HeLa PI positive. **b.** Pooled data (n=3 independent experiments) for % PI positive HeLa cells from the analysis such as that in **a** are shown (mean $\pm$ SEM). **c.** HeLa cells were treated with 125 µM H_2O_2 for 1 h prior to staining with 10 uM CellRox Green for 30 min and analysed by flow cytometry. Quantification of the fluorescence intensity relative to the untreated (UT) sample are shown representing the mean $\pm$ SEM (n=3 independent experiments). **d-e.** HeLa cells were treated with 100 µM DEM for 24 h and then stained immediately with propidium iodide for flow cytometric analysis of cell death. **d.** Representative dot plots of the indicated samples with the percentage of HeLa PI positive. **e.** Pooled data (n=3 independent experiments) for % PI positive HeLa cells from the analysis such as that in **d** are shown (mean $\pm$ SEM). **f.** HeLa cells were treated with 100 µM DEM for 24 h prior to staining and flow cytometry as in **c.** Quantification of the fluorescence intensity relative to the UT sample are shown representing the mean $\pm$ SEM (n=3 independent experiments). p values (two tailed student's t test) left to right top to bottom p = 0.6820, p = 0.0451, p = 0.9811, p = 0.0183. Source data are provided as a Source Data file.

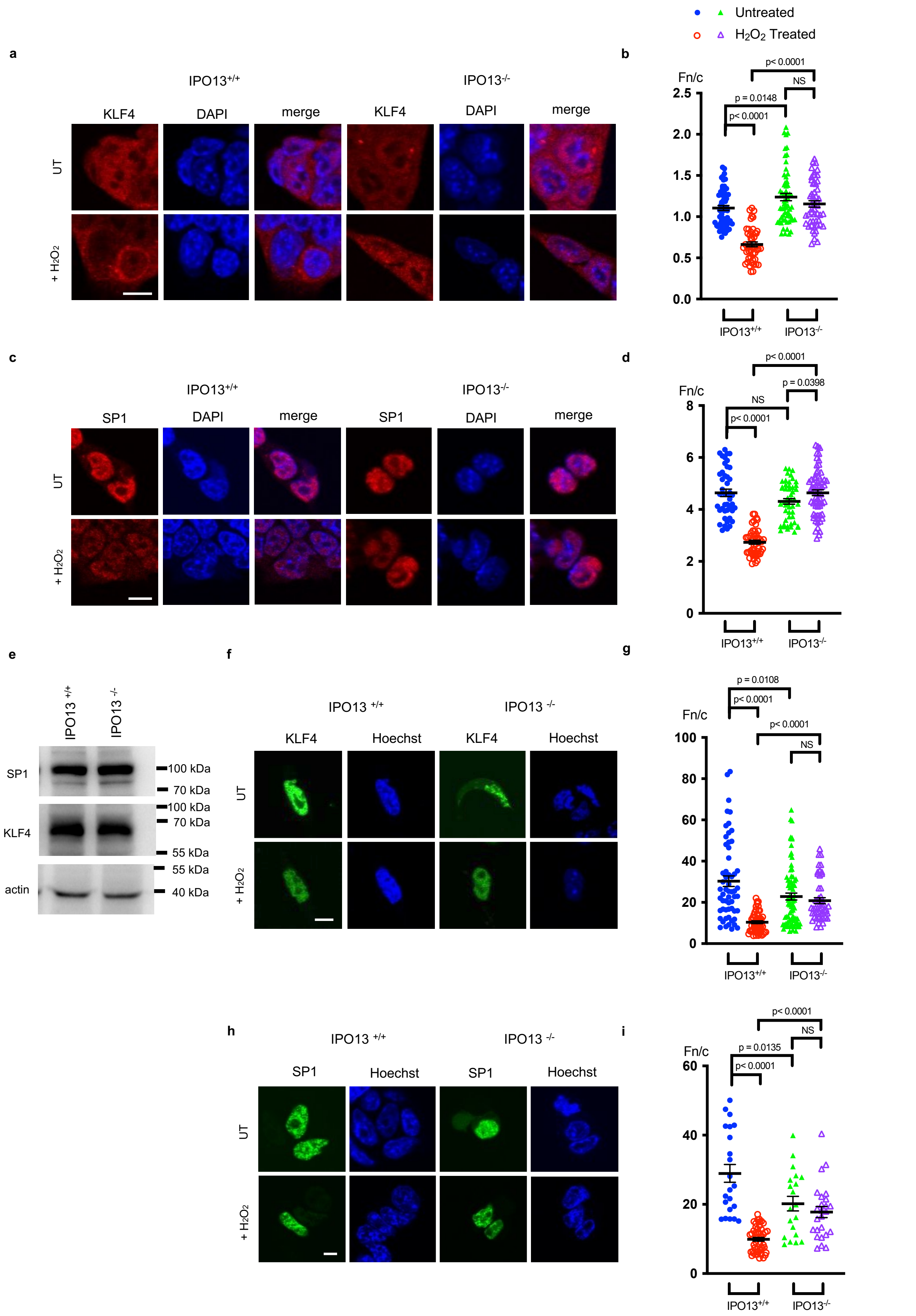

Supplementary Figure 8. IPO13 can mediate nuclear export of KLF4 and SP1 in response to H_2O_2 induced oxidative stress in ESCs. a-d. IPO13$^{+/+}$ and IPO13$^{-/-}$ cells were treated with 125 µM H_2O_2 for 1 h prior to processing for CLSM imaging. All cells were counterstained with DAPI to highlight nuclei. **a.** Representative images of ESCs immunostained with anti-KLF4. Scale bar = 10 µm **b.** Quantitative analysis of endogenous KLF4 protein localisation was carried out using the ImageJ software on images such as those in **a**, to determine the nuclear to cytoplasmic ratio (Fn/c). Data represent the mean $\pm$ SEM of the nuclear (Fn)

and cytoplasmic fluorescence (Fc) above background fluorescence of 56 cells ($IPO13^{+/+}$ Untreated (UT)), 47 cells ($IPO13^{+/+}$ + H_2O_2), 60 cells ($IPO13^{-/-}$ UT), 51 cells ($IPO13^{-/-}$ + H_2O_2). p values (two tailed student's t test) top to bottom: p < 0.0001, p = 0.1612, p = 0.0148 and p < 0.0001. **c.** Representative images of cells immunostained with anti-SP1. Scale bar = 10 µm **d.** Quantitative analysis of endogenous SP1 localisation carried out as per **b.** Data represent the mean ± SEM for 44 cells ($IPO13^{+/+}$ UT), 46 cells ($IPO13^{+/+}$ + H_2O_2), 49 cells ($IPO13^{-/-}$ UT), 63 cells ($IPO13^{-/-}$ + H_2O_2). p values (two tailed student's t test) top to bottom: p < 0.0001, p = 0.0398, p = 0.0632 and p < 0.0001. **e.** Total $IPO13^{+/+}$ and $IPO13^{-/-}$ ESC extracts were probed by Western blotting using rabbit-anti-SP1 (Milipore) or rabbit-anti-KLF4 (Abcam), with mouse-anti-actin (Abcam) as a control, and imaged using the ChemiDoc Gel Imaging System (Biorad), data representing n = 2 experiments **f-i.** $IPO13^{+/+}$ and $IPO13^{-/-}$ cells were transfected to express either GFP-tagged KLF4 (**f,g**) or GFP-tagged SP1 (**h,i**) and treated with 125 µM H_2O_2 for 1 hprior to CLSM imaging and counterstained with Hoechst. **f.** Representative images of cells ectopically expressing GFP-KLF4. Scale bar = 10 µm **g.** Quantitative analysis of ectopic KLF4 protein localisation performed as per **b**, representative of the mean ± SEM from 2 separate experiments of 58 cells ($IPO13^{+/+}$ UT), 54 cells ($IPO13^{+/+}$ + H_2O_2), 80 cells ($IPO13^{-/-}$ UT), 49 cells ($IPO13^{-/-}$ + H_2O_2). p values (two tailed student's t test) top to bottom: p = 0.0108, p < 0.0001, p < 0.0001 and p = 0.4051. **h.** Representative images of cells ectopically expressing GFP-SP1. Scale bar = 10 µm **i.** Quantitative analysis of ectopic SP1 protein localisation performed as per **b**, representative of the mean ± SEM of 33 cells ($IPO13^{+/+}$ UT), 33 cells ($IPO13^{+/+}$ + H_2O_2), 43 cells ($IPO13^{-/-}$ UT), 50 cells ($IPO13^{-/-}$ + H_2O_2). p values (two tailed student's t test) top to bottom: p < 0.0001, p = 0.3606, p = 0.0135 and p < 0.0001. Source data are provided as a Source Data file.

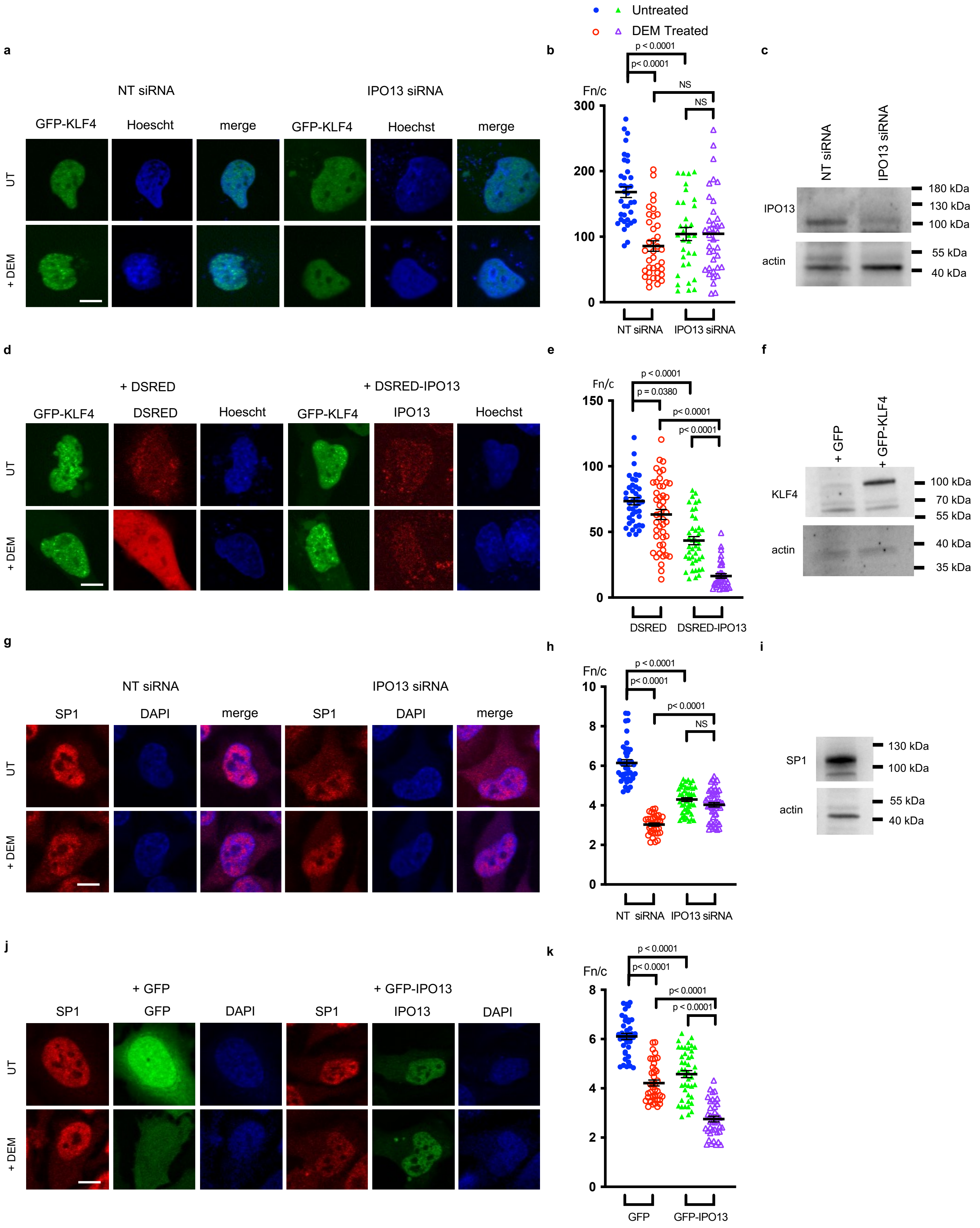

Supplementary Figure 9. IPO13 can mediate nuclear export of KLF4 and SP1 in response to DEM-induced oxidative stress. a-c. HeLa cells were transfected with non-targeting (NT) or IPO13 targeting siRNA 72 h prior to treatment with 100 μM DEM for 24 h. Cells were transfected to express GFP-KLF4 and processed for CLSM imaging and/or Western analysis. Cells were stained with Hoechst to highlight nuclei. **a.** Representative images of cells transfected to express GFP-KLF4. Scale bar = 10 μm **b.** Quantitative analysis of ectopic KLF4 protein localisation was carried out using the ImageJ software on images such as those in **a**, to determine the nuclear to cytoplasmic ratio (Fn/c). Data represent the mean + SEM from 2

separate experiments, where each experiment involved analysis of the nuclear (Fn) and cytoplasmic fluorescence (Fc) above background fluorescence. 36 cells (NT Untreated (UT)), 37 cells (NT + H_2O_2), 33 cells (IPO13 siRNA UT), 37 cells (IPO13 siRNA + H_2O_2) were analysed. p values (two tailed student's t test) top to bottom: $p < 0.0001$, $p < 0.0001$, $p = 0.1508$ and $p = 0.9813$. **c.** Total cell extracts were subjected to Western analysis using rabbit-anti-IPO13 (Protein Tech), with mouse-anti-actin (Abcam) as a control, and imaged using the ChemiDoc Gel Imaging System (Biorad). **d-e.** HeLa cells were treated with 100 μM DEM for 24 h and co-transfected to express either DSRED or DSRED-tagged IPO13 with GFP-KLF4 and nuclei stained with Hoechst prior to CLSM imaging. **d.** Representative images of cells co-expressing DSRED or DSRED-IPO13 with GFP-KLF4. Scale bar = 10 μm **e.** Quantitative analysis of ectopic KLF4 protein localisation performed as per **c**, representative of the mean ± SEM of 40 cells (DSRED UT), 46 cells (DSRED + H_2O_2), 41 cells (DSRED-IPO13 UT) and 35 cells (DSRED-IPO13 + H_2O_2). p values (two tailed student's t test) top to bottom: $p < 0.0001$, $p = 0.0380$, $p < 0.0001$ and $p < 0.0001$. **f.** Total cell extracts from HeLa transfected with GFP or GFP-KLF4 were probed as in **c**, with rabbit-anti-KLF4 (Abcam) and mouse-anti-actin (Abcam) as a control, representing n = 1 experiment. **g-i.** HeLa cells were transfected with siRNA as in **a-c.** prior to treatment with 100 μM DEM for 24 h. Cells immediately processed for CLSM imaging and/or Western analysis. Cells were counterstained with DAPI. **g.** Representative images of cells immunostained with anti-SP1(Milipore). Scale bar = 10 μm **h.** Quantitative analysis of endogenous SP1 localisation carried out as per **b**. representing the mean ± SEM of 41 cells (NT UT), 36 cells (NT + H_2O_2), 47 cells (IPO13 siRNA UT), 49 cells (IPO13 siRNA + H_2O_2). p values (two tailed student's t test) top to bottom: $p < 0.0001$, $p < 0.0001$, $p < 0.0001$ and $p = 0.0666$. **i.** Total cell extracts were probed by Western blotting a in **c.** using ant-SP1 (Millipore) and mouse-anti-actin (Abcam) as a control. **j-k.** HeLa cells were treated with 100 μM DEM for 24 h and co-transfected to express either GFP or GFP-tagged IPO13 prior to being processed for CLSM imaging. Cells are counterstained with DAPI. **j.** Representative images of cells immunostained with anti-SP1. Scale bar = 10 μm **k.** Quantitative analysis of endogenous SP1 localisation carried out as in **c**. with data representative of the mean ± SEM of 39 cells (GFP UT), 41 cells (GFP + DEM), 45 cells (GFP-IPO13 UT), 39 cells (GFP-IPO13 + DEM). p values (two tailed student's t test) top to bottom: $p < 0.0001$, $p < 0.0001$, $p < 0.0001$ and $p < 0.0001$. Source data are provided as a Source Data file.

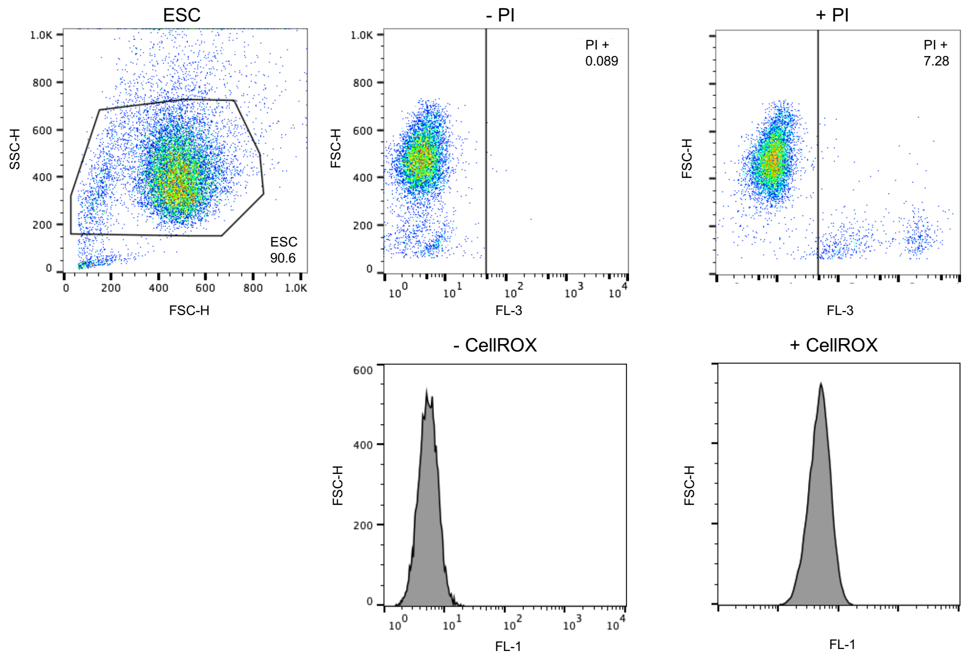

Supplementary Figure 10. Gating strategy to identify cell death and reactive oxygen species in mouse embryonic stem cells. IPO13$^{+/+}$ and IPO13$^{-/-}$ mouse embryonic stem cells were gated to remove debris (FSC/SSC) and unstained cells were used to gate for PI (propidium iodide) positive cells or CellROX positive cells.

Supplementary Table 1. Oligonucleotides used for qPCR Analysis

Primer Name	Forward Sequence	Reverse Sequence
Cyp2s1	TGGCACAGGAGAAACAAGAC	ACATACCTGCTGTTTGCTGG
Ddit3	CATCCCCAGGAAACGAAGAG	TCTTCCTCCTGGGCCATAGA
Dnajb5	ACGCCGAGGAGAAGTTTAAG	GAGGCCTTCCTCACCATACT
Dok1	GAGGCTTCTGAACGCTGCGGCTTGC	AGAACATTACCTTGTCCCGGCCAT
Fosb	CCGAGAAGAGACACTTACCC	CTCTTCAAGCTGATCAGTTTCC
Grxcr2	CCATAAGCCTCCACCCATTA	CCCTGTCTCCATCATTCTCC
Hspa1a	ATGGACAAGGCGCAGATCC	CTCCGACTTGTCCCCCAT
Il5	TTGACAAGCAATGAGACGATGAG	ACCAGTTTGAGGCCAGCCTGCG
Lef1	GAGCACTTTTCTCCGGGATC	GGGATTTCAGGAGCTGGAG
Map3k14	AGAAGACCGAGCCCTTTACTACCT	ACAGGAGCACGTTGTCAGCTTTGA
Neu3	CTTCAGGCCTTCTTCCGAGA	GAGTAGGGTGGGACTTCCTC
Ppard	TCCATCGTCAACAAAGACGGG	ACTTGGGCTCAATGATGTCAC
Rasd1	CCACACAACCTGAGGACCTT	TTCACAGCAGGTGACTGTCC
Rnu12	AACTTATGAGTAAGGAAAATAACGATTCG	AATTTTTGAGCGGGTATAGG
Rreb1	CACCACAGATACCAACAAGTTCAGTCC	CTTCTCAGGGAGCAGGTGGTAACTCC
Sdha	GCTCCTGCCTCTGTGGTTGA	AGCAACACCGATGAGCCTG
Snai3	TGCCGCGCTCCTTCCTGGTG	CAGAGGTACTGTCCCAAGGC
Stox1	AACACCTTGAAGGGACAGAGAA	ATCCAAAATGGCGGAATTAGTA
Tbp	GAAGAACAATCCAGACTAGCAGCA	CCTTATAGGGAACTTCACATCACAG
