# Supplementary material for: Nuclear transporter Importin-13 plays a key role in the oxidative stress transcriptional response
Source: Nat Commun. 2021 Oct 8;12:5904. doi: 10.1038/s41467-021-26125-x (PMC8501021; doi:10.1038/s41467-021-26125-x)
Supplement: Supplementary file 3 — Description of Additional Supplementary Files [file 41467_2021_26125_MOESM3_ESM.pdf]

Description of Additional Supplementary Files

Supplementary Data 1: DEGs for KO NS relative to WT NS ($IPO13^{-/-}$ relative to $IPO13^{+/+}$)

Supplementary Data 2: DEGs for WT ST relative to WT NS ($IPO13^{+/+}$ + H2O2 relative to $IPO13^{+/+}$ NS)

Supplementary Data 3: DEGs for KO ST relative to KO NS ($IPO13^{-/-}$ + H2O2 relative to $IPO13^{-/-}$ NS)
