# Supplementary material for: Nuclear transporter Importin-13 plays a key role in the oxidative stress transcriptional response
Source: Nat Commun. 2021 Oct 8;12:5904. doi: 10.1038/s41467-021-26125-x (PMC8501021; doi:10.1038/s41467-021-26125-x)
Supplement: Supplementary file 7 — Reporting Summary [file 41467_2021_26125_MOESM7_ESM.pdf]

nature portfolio

Corresponding author(s): David A. Jans

Last updated by author(s): 20/08/21

Reporting Summary

Nature Portfolio wishes to improve the reproducibility of the work that we publish. This form provides structure for consistency and transparency in reporting. For further information on Nature Portfolio policies, see our Editorial Policies and the Editorial Policy Checklist.

Statistics

For all statistical analyses, confirm that the following items are present in the figure legend, table legend, main text, or Methods section.

n/a	Confirmed	
☐	☒	The exact sample size (n) for each experimental group/condition, given as a discrete number and unit of measurement
☐	☒	A statement on whether measurements were taken from distinct samples or whether the same sample was measured repeatedly
☐	☒	The statistical test(s) used AND whether they are one- or two-sided<br>*Only common tests should be described solely by name; describe more complex techniques in the Methods section.*
☐	☒	A description of all covariates tested
☐	☒	A description of any assumptions or corrections, such as tests of normality and adjustment for multiple comparisons
☐	☒	A full description of the statistical parameters including central tendency (e.g. means) or other basic estimates (e.g. regression coefficient) AND variation (e.g. standard deviation) or associated estimates of uncertainty (e.g. confidence intervals)
☐	☒	For null hypothesis testing, the test statistic (e.g. F, t, r) with confidence intervals, effect sizes, degrees of freedom and P value noted<br>*Give P values as exact values whenever suitable.*
☒	☐	For Bayesian analysis, information on the choice of priors and Markov chain Monte Carlo settings
☒	☐	For hierarchical and complex designs, identification of the appropriate level for tests and full reporting of outcomes
☒	☐	Estimates of effect sizes (e.g. Cohen's d, Pearson's r), indicating how they were calculated

Our web collection on statistics for biologists contains articles on many of the points above.

Software and code

Policy information about availability of computer code

Data collection	ChemiDoc Imager (Biorad), Olympus FV1000 IX81 invert confocal microscope, Illumina HiSeq2000, 384 well rtPCR (Biorad), FACS Calibur (BD Bioscience), CellQuest (Version 3.3).
Data analysis	Image Lab (version 6.0), Fiji software (version 2.1.0/1.53c), Image J (version 1.48), Prism (version 8), Voom/Limma Degust (v.0.20), , Biorad CFX Manager 3.1, Flowjo (version 10.7.2).

For manuscripts utilizing custom algorithms or software that are central to the research but not yet described in published literature, software must be made available to editors and reviewers. We strongly encourage code deposition in a community repository (e.g. GitHub). See the Nature Portfolio guidelines for submitting code & software for further information.

Data

Policy information about availability of data

All manuscripts must include a data availability statement. This statement should provide the following information, where applicable:

- Accession codes, unique identifiers, or web links for publicly available datasets
- A description of any restrictions on data availability
- For clinical datasets or third party data, please ensure that the statement adheres to our policy

The data that support this work are available from the corresponding author upon reasonable request. Unprocessed blots are provided with this paper. The RNA sequencing data generated in this study has been submitted to the Gene Expression Omnibus (GEO) database under accession number 'GSE108913' [https://www.ncbi.nlm.nih.gov/geo/query/acc.cgi]. Database and tools used in this study are publicly available; RSAT Peak-Motif [http://rsat.sb-roscoff.fr/peak-motifs_form.cgi], Trawler [https://trawler.erc.monash.edu.au], MEME-ChIP [https://meme-suite.org/meme/tools/meme-chip], STAMP [http://www.benoslab.pitt.edu/stamp/], JASPAR [http://jaspar.genereg.net], GREAT [http://great.stanford.edu/public/html/index.php], Galaxy [https://usegalaxy.org],

DAVID [https://david.ncifcrf.gov] and UCSC Mouse Encode (Track Search, Table Browser) [https://genome.ucsc.edu/ENCODE/].

Field-specific reporting

Please select the one below that is the best fit for your research. If you are not sure, read the appropriate sections before making your selection.

- [x] Life sciences
- [ ] Behavioural & social sciences
- [ ] Ecological, evolutionary & environmental sciences

For a reference copy of the document with all sections, see nature.com/documents/nr-reporting-summary-flat.pdf

Life sciences study design

All studies must disclose on these points even when the disclosure is negative.

Sample size	Number of cells used for quantitative and statistical analyses were above 25 to ensure the representative images shown are typical results. Consistent results were observed from randomly picked 6-7 cells , therefore 25 cells in total were consider enough. For FACS-based experiments, a standard number off at least 15,000 cells/sample was analysed, producing consistent results to give confidence in accurate results. Co IP were replicated twice and three times to verify interactions were reproducible.
Data exclusions	For Fn/c analysis of imaging experiments, the 10 percent highest and lowest values (outliers) were excluded to account for any cells where localisation of a protein differed greatly from the rest of the cells and was not reflective of the cell population. This criteria is established prior to performing the experiment.
Replication	Experimental findings were replicated through independent biological replicates (stipulated as n-number associated in figure legend) or where indicated through technical replicate (n- number associated in figure legend).
Randomization	Cells in each experiment were derived from the same pool of parent cells. Cells were randomly assigned to treatment or control groups.
Blinding	For imaging experiments in cells and other in vitro experiments, cells were selected for imaging using the DAPI channel to reduce any bias. Generally, blinding was not relevant as the reported data was based on quantitative measurements.

Reporting for specific materials, systems and methods

We require information from authors about some types of materials, experimental systems and methods used in many studies. Here, indicate whether each material, system or method listed is relevant to your study. If you are not sure if a list item applies to your research, read the appropriate section before selecting a response.

Materials & experimental systems

n/a	Involved in the study	
[ ]	[x]	Antibodies
[ ]	[x]	Eukaryotic cell lines
[x]	[ ]	Palaeontology and archaeology
[x]	[ ]	Animals and other organisms
[x]	[ ]	Human research participants
[x]	[ ]	Clinical data
[x]	[ ]	Dual use research of concern

Methods

n/a	Involved in the study	
[x]	[ ]	ChIP-seq
[ ]	[x]	Flow cytometry
[x]	[ ]	MRI-based neuroimaging

Antibodies

Antibodies used	Rabbit polyclonal anti-SP1 (Cat no. ABE135 Millipore), anti-IPO13 (Cat no. 11696-2-AP, Protein Tech), anti-KLF4 (Cat no. ab75486 and ab129473, Abcam) antibodies, anti-EIF1A (Cat no. 38976, Abcam) and mouse monoclonal anti-NFAT1 (Cat no. ab2722, Abcam), anti-Actin (Cat no. ab3280, Abcam), anti-GFP (Cat no. 11814460001, Roche) and ant-UBC9 (Cat no. 610749, BD) antibodies were used as indicated. Alexa-Fluor 568-goat anti-rabbit (A11036), Alexa-Fluor 568-goat anti-mouse (A11004), Alexa-Fluor 488-goat anti rabbit (A11008) and Alexa-Fluor 488-goat anti mouse (A11001) IgG secondary antibodies were from Invitrogen.
Validation	Antibodies were previously validated as per the manufacturer's website or published papers cited in this manuscript. Briefly, rabbit polyclonal anti-SP1 (Cat no. ABE135 Millipore) was evaluated by Western Blotting in HeLa nuclear extract, rabbit polyclonal anti-IPO13 (Cat no. 11696-2-AP, Protein Tech) was validated in HeLa by IF and in MCF-7, Neruo-2a and mouse lung by Western blot, rabbit polyclonal anti-KLF4 (ab129473, Abcam) was tested for application in Western blot and IF, polyclonal anti-EIF1A (ab38976) was tested using western blot in HeLa, Jurkat and A-431 cell lines and for IF in HeLa, mouse monoclonal anti-NFAT1 (Cat no. ab2722, Abcam) was tested for use in Flow cytometry,Western blot and IF, mouse monoclonal anti-actin (ab3280) was validated with Western blot in NIH 3T3, MDA-MB-231 and HeLa cell lines and with IF in HeLa cells, mouse anti-UBC9 (Cat no. 610749, BD) was tested in development for use in Western blot, IHC, and IF. Anti-GFP is tested for functionality and purity relative to a reference standard to confirm the quality of each new reagent preparation, tested for application in western blot and immunoprecipitation.

Eukaryotic cell lines

Policy information about cell lines

Cell line source(s)	Hela cells (from ATCC) and ES Cells generated at Children's Medical Research Institute (described in reference 4)
Authentication	None of the cell lines used were authenticated
Mycoplasma contamination	Cell lines routinely tested negative for mycoplasma contamination.
Commonly misidentified lines (See ICLAC register)	Not included

Flow Cytometry

Plots

Confirm that:

- [x] The axis labels state the marker and fluorochrome used (e.g. CD4-FITC).
- [x] The axis scales are clearly visible. Include numbers along axes only for bottom left plot of group (a 'group' is an analysis of identical markers).
- [x] All plots are contour plots with outliers or pseudocolor plots.
- [x] A numerical value for number of cells or percentage (with statistics) is provided.

Methodology

Sample preparation	Cells for FACS analysis were trypsinised and washed with PBS before being resuspended in ice cold PBS. Cells were stained with 1ug/ml propidium iodide to measure for dead cells immediately prior to analysis. CellROX Green was used to measure abundance of reactive oxygen species in cells, added at 5 or 10 uM, 30 min prior to analysis.
Instrument	FACS Calibur
Software	CELLQuest Software Version 3.3
Cell population abundance	A minimum of 15,000 cells were recorded by the cytometer for each sample.
Gating strategy	IPO13+/+ and IPO13-/- mouse embryonic stem cells or HeLa were gated to remove debris (FSC/SSC) and unstained cells were sued to gate for PI positive cells (FL3 channel). Alternatively, unstained cells were used to gate for CellROX Green (FL1 Channel).

- [x] Tick this box to confirm that a figure exemplifying the gating strategy is provided in the Supplementary Information.
