# Supplementary material for: Nuclear transporter Importin-13 plays a key role in the oxidative stress transcriptional response
Source: Nat Commun. 2021 Oct 8;12:5904. doi: 10.1038/s41467-021-26125-x (PMC8501021; doi:10.1038/s41467-021-26125-x)

Figure 5a

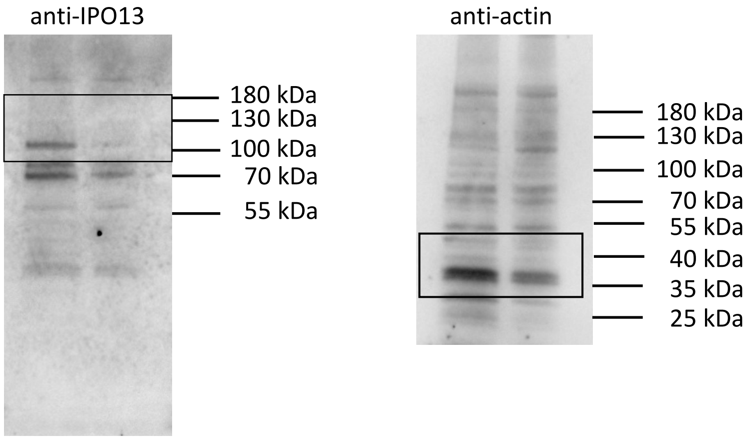

Figure 6a

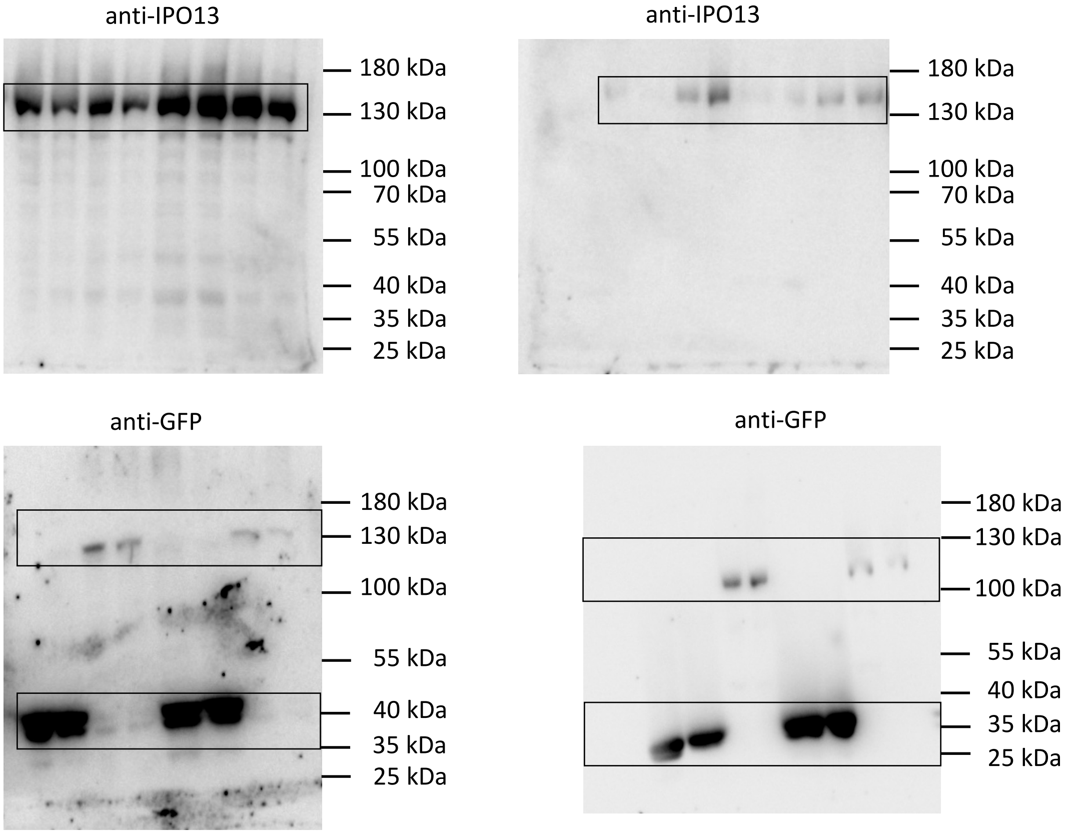

Figure 6b

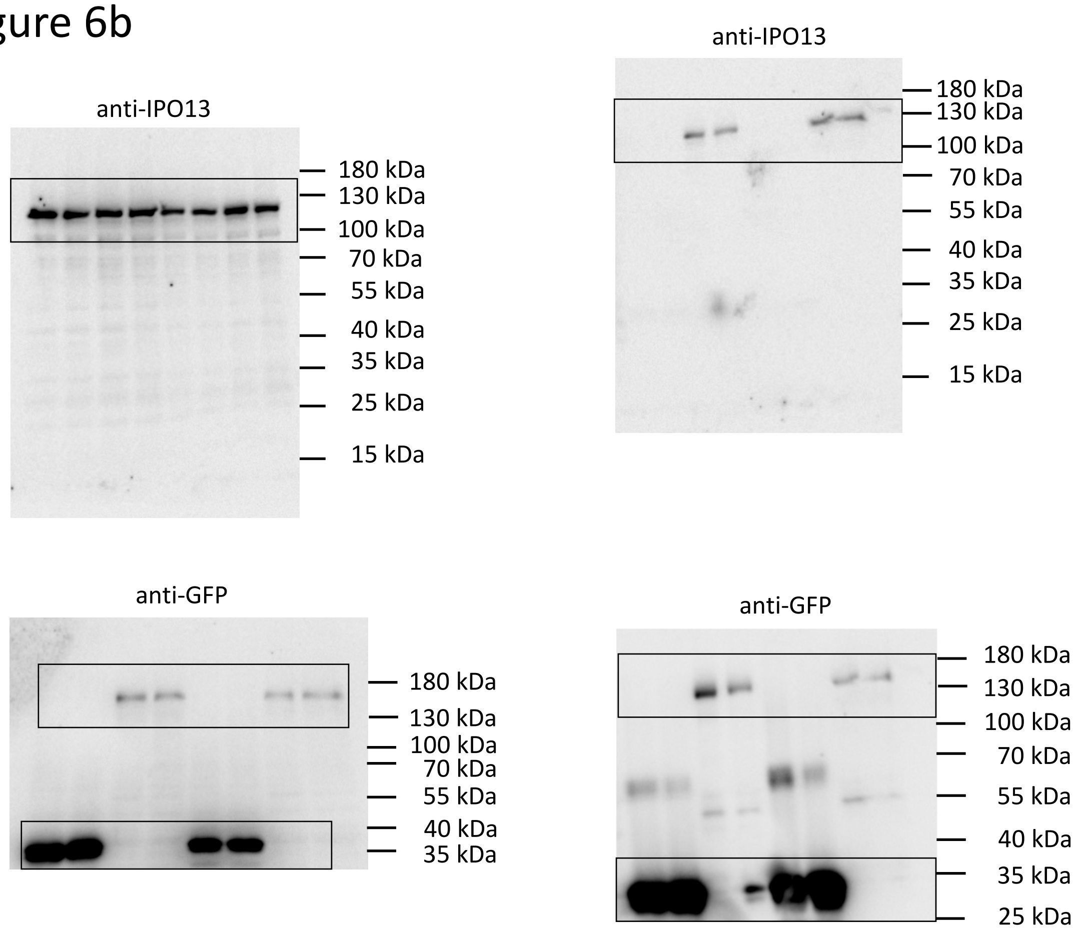

anti-IPO13
180 kDa
130 kDa
100 kDa
70 kDa
55 kDa
40 kDa
35 kDa
25 kDa
15 kDa
anti-IPO13
180 kDa
130 kDa
100 kDa
70 kDa
55 kDa
40 kDa
35 kDa
25 kDa
15 kDa
anti-GFP
180 kDa
130 kDa
100 kDa
70 kDa
55 kDa
40 kDa
35 kDa
anti-GFP
180 kDa
130 kDa
100 kDa
70 kDa
55 kDa
40 kDa
35 kDa
25 kDa

Supplementary Figure 3e

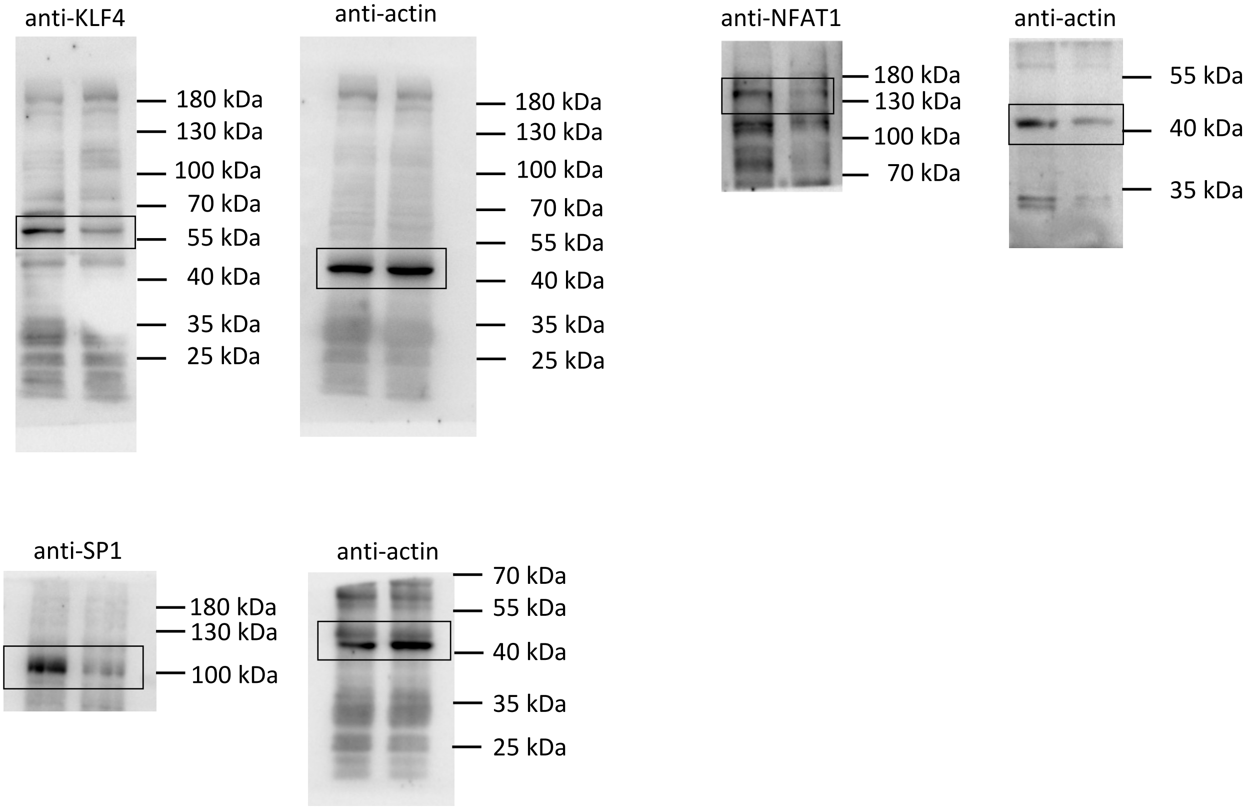

Supplementary Figure 6e

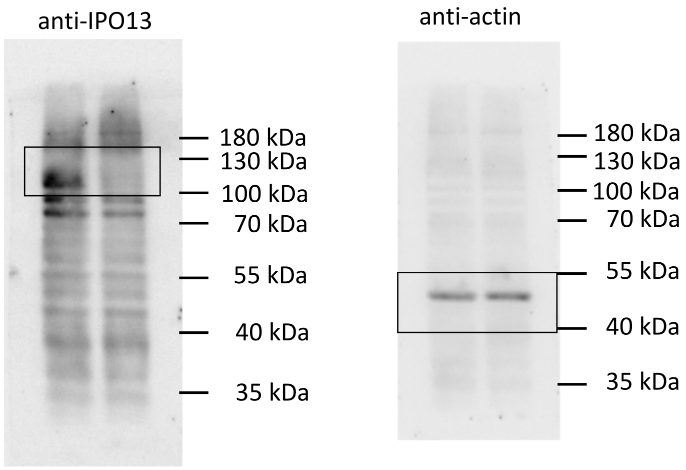

Supplementary Figure 6j

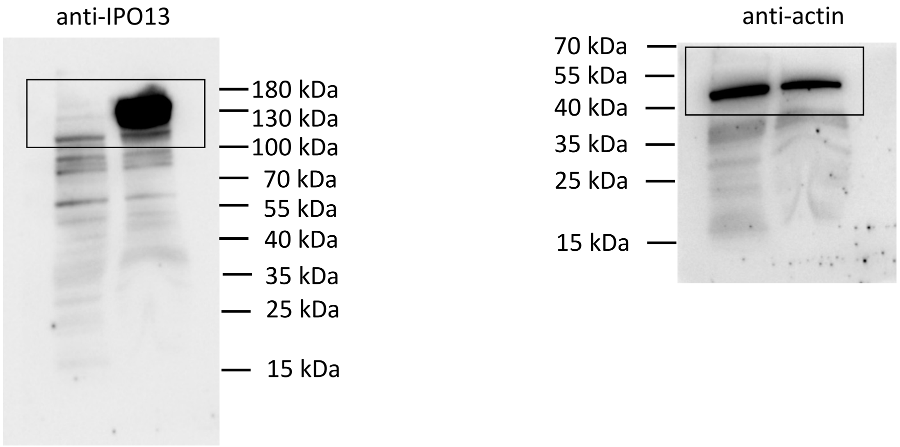

Supplementary Figure 6o

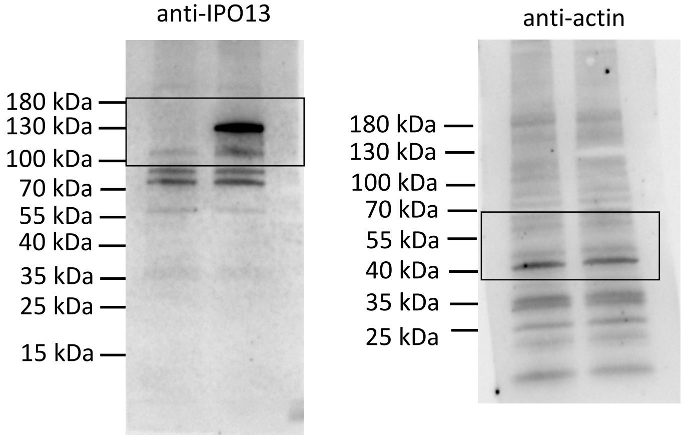

Supplementary Figure 8e

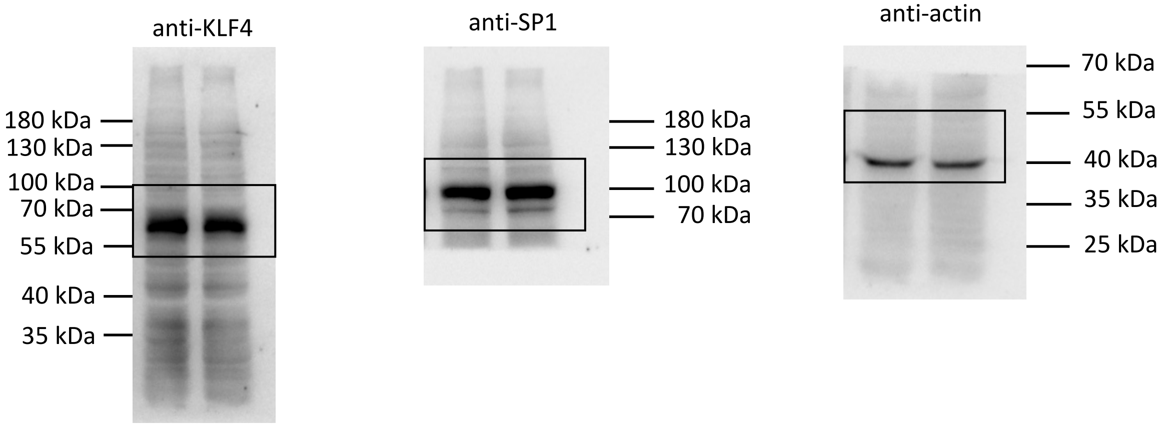

Supplementary Figure 9c

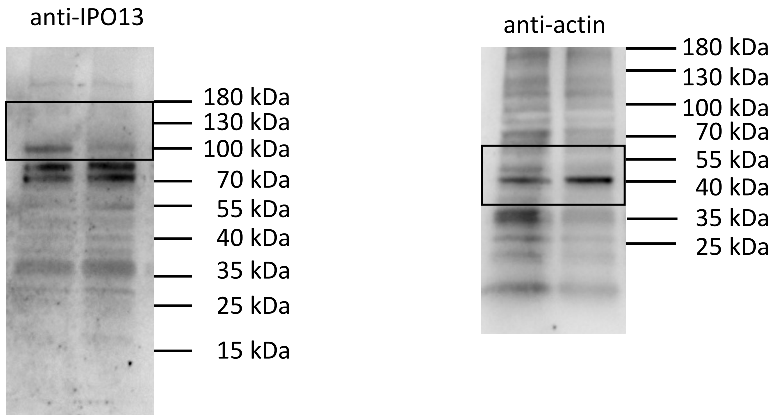

Supplementary Figure 9f

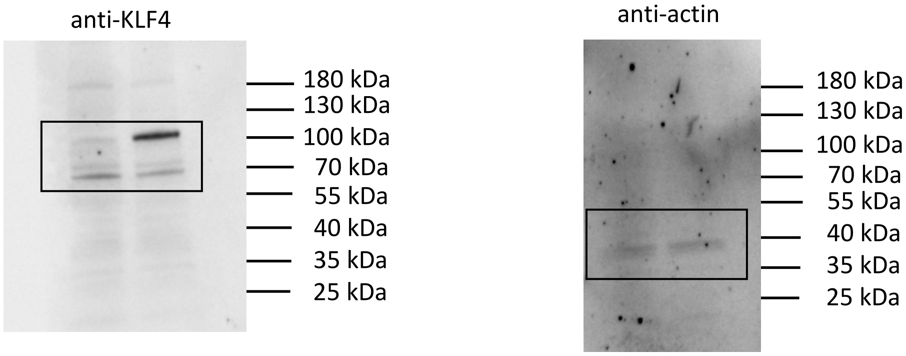

Supplementary Figure 9i

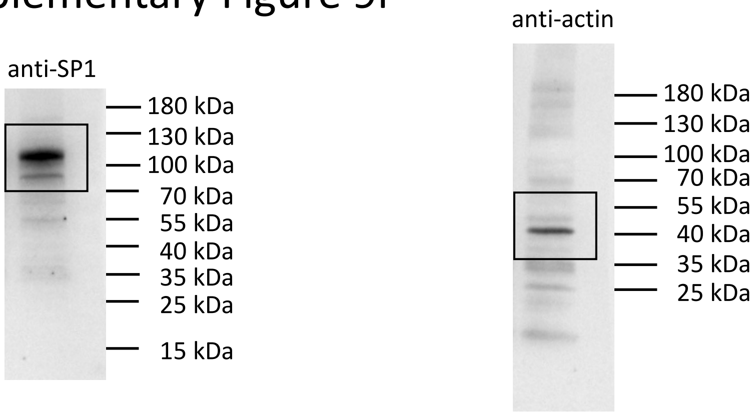

Supplement: Supplementary file 8 — Source Data [file 41467_2021_26125_MOESM8_ESM.zip › Unprocessed Blots Source Data.pdf]
